# Polyurethane Application to Transform Screen-Printed Electrode for Rapid Identification of Histamine Isolated from Fish

**DOI:** 10.1155/2023/5444256

**Published:** 2023-04-03

**Authors:** Muhammad Abdurrahman Munir, Jamia Azdina Jamal, Mazlina Mohd Said, Sofian Ibrahim, Mohamad Syahrizal Ahmad

**Affiliations:** ^1^Department of Pharmacy, Faculty of Health Sciences, Alma Ata University, 55184 Bantul, Yogyakarta, Indonesia; ^2^Drug and Herbal Centre, Faculty of Pharmacy, National University of Malaysia, Jalan Raja Muda Abdul Aziz, Kuala Lumpur 50330, Malaysia; ^3^Malaysian Nuclear Agency, 43000 Kajang, Bangi, Malaysia; ^4^Faculty of Science and Mathematics, Universiti Pendidikan Sultan Idris, Tanjung Malim 35900, Malaysia

## Abstract

The toxicity of histamine has attracted numerous researchers to develop a method for histamine determination purposes. The Food and Drug Administration (FDA) unequivocally prohibits the consumption of histamine above 50 mg·kg^−1^. Thus, an innovation in histamine detection in fish has been developed in this research. The investigation of the histamine level in fish has been conducted by using an electrochemical sensor approach and producing a polymer via molecularly imprinted polymer (MIP) on a screen-printed electrode. The technique was validated by assessing the shifts in electron shifting using the cyclic voltammetry (CV) approach and electrochemical impedance spectroscopy (EIS), whereas differential pulse voltammetry (DPV) was applied to validate the sensor method. The instruments showed a linear response ranging from 1–1000 nmol·L^−1^, with a detection limit of MIP/SPE at 1.765 nmol·L^−1^ and 709 nmol·L^−1^ for the NIP/SPE, respectively. The sensing technique was employed to determine the histamine level in selected samples at room temperature (25°C). The outcomes of this study indicated that the validated chemical sensor allowed accurate and precise detection of fish samples and can be categorized as a simple approach. The instrument is inexpensive and suitable for on-site detection.

## 1. Introduction

The safety of food has become an imperative issue that must be handled properly owing to the ease with which food can be contaminated in the food chain system [1]. A statement has been reported by the World Health Organization (WHO) that one in ten people gets food poisoning and almost half a million die from food poisoning [2]. Even in Southeast Asia, particularly in Indonesia and Malaysia, food poisoning causes 22.8 million cases of diarrhea and 37,600 fatalities [3]. Furthermore, the food must be secured, and monitoring the food is imperative, both globally and in the Southeast Asia region [4]. Among various foods that have been spread all over the world, aquatic foods have been related to food security issues owing to the massive consumption of aquatic food products in Southeast Asia [5, 6]. It is very common that physicochemical reactions can occur during the storage of food. The reactions can be influenced by several factors, such as heat, light, the packaging process, and moisture; furthermore, the addition of food adulterants can lead to the alteration of food content and cause health threats to humans [7].

Aquatic food products have a high nutritional value that is very useful for humans; however, compared to land animals, they contain more oils and fats, which cause several issues such as obesity, diabetes, and cardiovascular problems [8]. Among all kinds of aquatic foods, fish is the best choice, according to nutrition experts; it also has low fat and high protein [9].

Nevertheless, the lucrativeness of fish may change into a problem owing to the decarboxylation process that occurs inside the fish body, producing biogenic amines. During the decarboxylation process, the amino acids in fish muscle become biogenic amines. The most popular biogenic amine, and a hot topic among researchers, is histamine. The consumption of histamine may lead to food toxicity, also known as histamine poisoning. Several effects may occur owing to the presence of histamine inside the bloodstream, and various symptoms occur, ranging from mild to even causing death [10, 11].

As a consequence, several countries have issued regulations for fish consumption that contain histamine. The United States of America (USA), through the Food and Drug Administration (FDA), only allowed 50 mg·kg^−1^ of histamine, whereas the European Union (EU) has set the histamine level between 100 and 200 mg·kg^−1^ [12]. Moreover, food poisoning from histamine is commonly related to several particular aquatic animals, such as sardine, mackerel, tuna, anchovy, and herring. Moreover, the development of a cheap and fast technique for histamine detection is imperative [13].

Numerous approaches have been performed to determine histamine, and most of them are chromatographic-based. However, there are several issues before using these methods, such as long-term analysis, complex instruments, and the need for derivatizing agents to derivatize histamine and increase the sensitivity of HPLC [14, 15], whereas the application of gas chromatography (GC) is restricted to laboratory facilities and complex preprocessing methods and usually requires a derivatization process, which is unsuitable for daily and on-site detection of histamine [16, 17]. The enzyme-linked immunosorbent assay (ELISA) offers a specific method for histamine detection, but this technique requires a specific environment to maintain detection precision, accuracy, and stability [18].

The colorimetric method becomes a better choice compared to the conventional methods, such as the straightforward technique, because it is fast and has attracted numerous researchers to study it [19–21]. Nevertheless, there are restrictions to this method, such as the accuracy being very low, the extinction coefficient being insufficient, and the color resolution being low, which cause inaccurate estimation [22, 23].

Furthermore, biosensors for histamine detection are an alternative approach, where this technique combines a bioreceptor with a transduction scheme. Generally, biosensors offer several advantages, such as rapid response, low cost, ease of operation, and high selectivity and accuracy [24, 25]. The bioreceptors generally employed for biosensors are antibody or enzyme materials, and they have satisfactory selectivity [26, 27]. However, they are also very expensive and have low stability caused by several factors such as pH, ionic content, humidity, and high temperature [28]. As a solution for receptor material, there is specific material such as molecularly imprinted material (MIP) that offers several superiorities such as stability at high temperatures, robustness, high reproducibility, and on-site application [29]. Generally, MIP is a synthetic material produced by cross-linking monomers with polymerizing functions [30, 31]. Furthermore, the MIP properties propose satisfactory stability, easy production, a low budget, and robustness [32].

Electrochemical sensor approaches have a specific regulation that is related to the electrical parameters to build on the electrode surface. The specification of the sensor must be fulfilled to produce a satisfactory reproducible material and, consequently, obtain a satisfactory sensing instrument. Nevertheless, the process is not straightforward when the analyte experiences oxidation below its potential values [33], particularly histamine. Therefore, there are several MIP materials for histamine detection [34]. Thus, according to our findings, there is only one study that produces the MIP by electropolymerization [35].

This study conveys an electrochemical sensor for histamine detection on-site for the first time through the combination of MIP material with electropolymerized polyurethane (PU). PU is a unique polymer that has several advantages, such as good stability, being easily produced, being inexpensive, and having sufficient electrical properties that can be applied for histamine detection using the electrochemical sensor approach [36]. Furthermore, the MIP film was acquired by choosing the best states to develop a polyurethane-based imprinted film. The validated chemical sensor was validated and verified and then used to analyze histamine in selected samples.

## 2. Materials and Methods

### 2.1. Instruments

Electrochemical assessments were applied by a Metrohm Autolab Electrochemical Workstation provided by the Faculty of Science and Technology, Universiti Kebangsaan Malaysia. The SPE was a working electrode, while the reference and auxiliary electrodes were the Ag/AgCl with a double-junction system and platinum wire, respectively.

### 2.2. Reagents

All reagents were applied without purification. Histamine (His) (≥99%) was purchased from Sigma Aldrich Sdn. Bhd. Cadaverine (Cad), putrescine (Put), tyramine (Tyr), heptylamine (Hep), and spermidine (Spe) were bought at 99% purity and purchased from Sigma Aldrich Sdn. Bhd. Potassium hexacyanoferrate III (K_3_ [Fe(CN_6_]) and potassium hexacyanoferrate II-3-hydrate (K_4_ [Fe(CN_6_] 3H_2_O) were obtained from Merch Sdn. Bhd. Sulfuric acid (H_2_SO_4_), ≥97%, and lithium perchlorate were purchased from Sigma Aldrich, and polyurethane (PU) was produced by Munir et al. [36]. The phosphate buffer solution (PBS) was obtained by the combination of sodium dihydrogen phosphate (Na_2_H_2_PO_4_) and disodium hydrogen phosphate (Na_2_HPO_4_), and both reagents were purchased from Merck, Sdn. Bhd. Ultrapure Milli-Q water was applied throughout.

### 2.3. Histamine Isolation from Fish Products

There was a fish applied in this study, namely, mackerel (*Rastrelliger kanagurta*). The application of fish in this study was to observe the accuracy and precision of this sensor approach. Approximately 2 g of fish samples were dissolved using trichloroacetic acid (TCA) (2.5%) in 10 mL and homogenized using a centrifuge. Afterward, a 20 mL centrifuge tube was applied to contain the homogenized sample, and TCA (2.5%) in 10 mL was added to the tube to degrade the proteins. To carry out the UAI method, the tubes were put in an ultrasonicator for 5 min at 50°C. After the sonication step, the tubes were cooled and centrifuged for 5 min at 2000 rpm. Finally, the mixture was filtered using a membrane filter (0.45 *µ*m pore size) and diluted in PBS (100 mmol·L^−1^) before being analyzed using an electrochemical sensor.

### 2.4. Solutions

All solutions were obtained using ultrapure water and kept at 4°C. The H_2_SO_4_ (0.1 M) was employed to purify the SPE, whereas the polyurethane (0.01 M) was combined with the lithium perchlorate (0.1 M) with the addition of histamine (100 nmol·L^−1^) to generate the MIP film that will undergo the polymerization mixture. Control was established using polyurethane (0.01 M) in lithium perchlorate (0.1 M), also known as a nonimprinted polymer (NIP). The behavior of histamine solution (100 nmol·L^−1^) and a combination solvent of cadaverine, putrescine, and histamine with a similar concentration (100 nmol·L^−1^), respectively, were applied to study the sensitivity and selectivity. The calibration curve of histamine standard solutions was organized in lithium perchlorate (0.1 M) ranging from 1–1000 nmol·L^−1^. The solutions of [Fe(CN)_6_]^4−^ and [Fe(CN)_6_]^3−^, 10 mM, respectively, were set up in PBS (100 mmol·L^−1^).

### 2.5. The Molecularly Imprinted Polymer (MIP) Preparation

**Figure 1:**
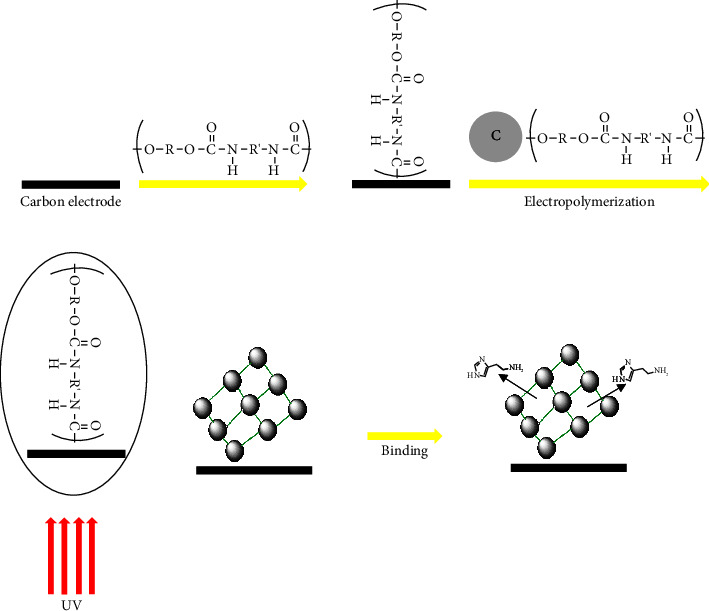
The MIP preparation for histamine determination.

The SPE as a working electrode was cleansed by electrochemical treatment by a cyclic voltammetry (CV) approach ranging from −0.1 to +1 at 1.5 V, scan rate (0.05 V), 10 cycles, in H_2_SO_4_ (0.1 M). Afterward, ultrapure water was applied to clean the SPE surface. The chronoamperometry method was applied at +0.70 V for 100 s to produce the molecularly imprinted polymer (MIP) by applying the polymerization mixture. To obtain this requirement, a metal-free photo atom transfer radical polymerization (ATRP) grafting step was produced using a pulsed UV laser as a light source to manufacture a thin MIP film on an electrode surface (see Figure 1).

### 2.6. FTIR Analysis

FTIR analysis was applied using a Perkin-Elmer Spectrum BX instrument using the diamond attenuation total reflectance (DATR) approach to verify the MIP. An FTIR spectroscopic analysis was executed at a wavenumber of 4000 to 600 cm^−1^ to verify the important peaks [37].

### 2.7. Electrochemical Procedures

There were three electrochemical measurements employed in this study, such as cyclic voltammetry (CV), electrochemical impedance spectroscopy (EIS), and differential pulse voltammetry (DPV). The CV was performed in this study to analyze the redox potential and the electrochemical reaction rates; the scanned potential ranged from −0.5 to +0.5 V (0.05 V·s^−1^), whereas the EIS was employed to study the conductivity of MIP, and the outcomes would be applied to establish the Randles equivalent circuit and determined by the Nyquist plots. Furthermore, several parameters were used, such as the frequency range of 0.1–100000 Hz and the sinusoidal wave at 0.01 V. These parameters are applied to reflect the kinetic reaction that occurs on the electrolyte-electrode interface and would be addressed as resistance, or Z′ (Ω), known as the specific part of the impedance, and the imaginary part, or −Z″ (Ω). Furthermore, the Nyquist plot contains a semicircle that would be used to estimate the charge-transfer resistance (*R*_ct_) [38]. Moreover, the last electrochemical measurement applied in this study, namely, the DPV method, was applied due to it being more selective and sensitive than the CV [39]; thus, the scanning potentials ranged from −0.1 to +0.9 V.

The response of the redox probe solution was obtained after the alteration occurred at the sensing surface electrode that is linked to the electrical properties. The detection limit (DL) (*x* + 3*σ*) was acquired from the linear response. The application of other biogenic amines in this study, such as cadaverine, tyramine, spermidine, heptylamine, and putrescine, was done to study the accuracy and precision of the method. Those biogenic amines are commonly discovered in fish products. Similar concentrations of histamine, cadaverine, tyramine, spermidine, heptylamine, and putrescine were applied in this study at 100 nmol·L^−1^. Furthermore, in this study, two devices were applied to determine the single histamine solution and the mixed solution of biogenic amines.

## 3. Results and Discussion

**Figure 2:**
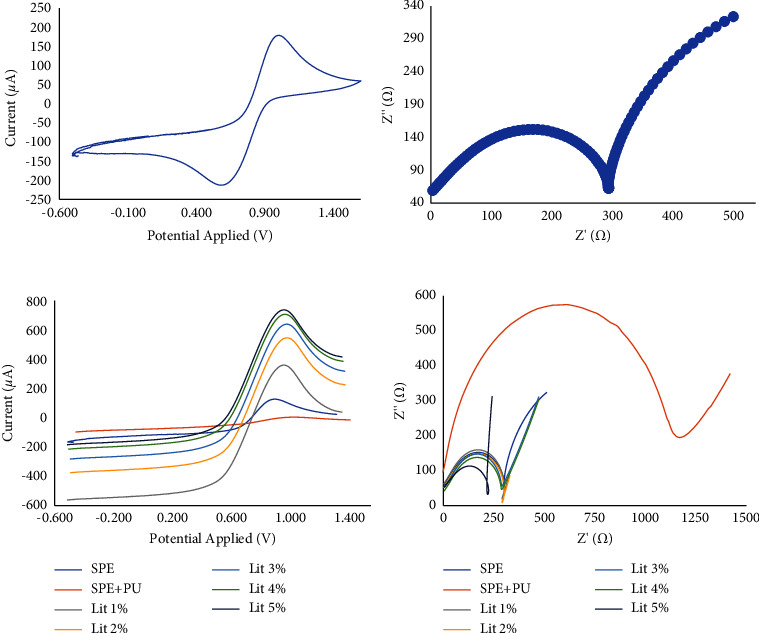
The study of an unmodified SPE using (a) CV and (b) EIS in PBS (100 mmol·L^−1^), and the study of unmodified and modified SPE using various concentrations of lithium perchlorate using (c) CV and (d) EIS in PBS (100 mmol·L^−1^).

The application of SPEs should be cleaned before use to determine the analyte; this is an imperative step to avoid interferences during the analysis. Thus, to ascertain the reproducibility of electrochemical sensors, several tests were employed, such as (i) using ethanol 98% to cleanse the electrode surface (3×), (ii) employing ethanol 98% to purify the electrode surface (3×), followed by the CV cleaning using H_2_SO_4_ (0.1 N) solution, and (iii) using merely a CV approach with the similar H_2_SO_4_ (0.1 N). The optimization of the potential applied and the duration of CV when cleansing treatment using the CV approach were studied, where the application of −0.1 to 1.5 V with 10 cycles was the satisfactory parameter. The EIS method was also applied beside the CV, and the plots of CV and EIS acquired under these parameters are presented in Figure 2. The SPE was purified and followed by the modification to produce an amine layer; afterwards, the MIP was generated. The production of the amine layer was influenced by the PU electropolymerization through its aromatic groups; furthermore, the MIP film was stably attached to the SPE surface [40]. Lithium perchlorate was applied to increase the conductivity of PU, and as expected, the addition of lithium perchlorate at various concentrations (1, 2, 3, 4, and 5%) influenced an *R*_ct_ increase, as shown in Figures 2(b) and 2(c).

**Figure 3:**
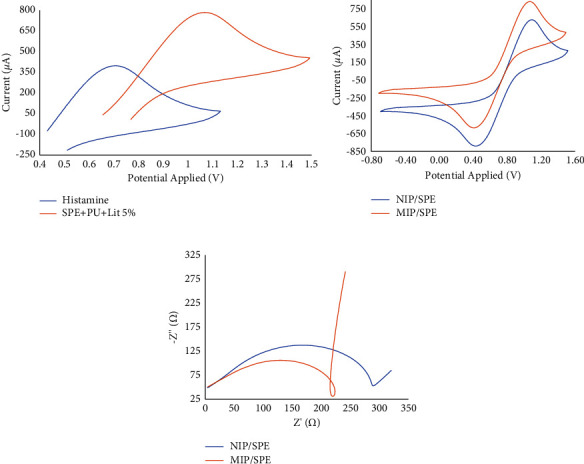
(a) Cyclic voltammograms of the modified electrode without histamine (SPE + PU + Lit 5%) and modified electrode that contains histamine to examine the potential application of histamine in 100 mmol·L^−1^ PBS; (b) the cyclic voltammograms of NIP/SPE and MIP/SPE using lithium perchlorates (5%) in 100 mmol·L^−1^ PBS; and (c) the spectra of the impedance of NIP/SPE and MIP/SPE in PBS (100 mmol·L^−1^).

The characteristics of PU and histamine (50 nmol·L^−1^) were first examined using an electrochemical approach in solo and mingled solutions to avoid the limitation that occurs. By employing the identical potential ranges, the first examination ensured that histamine and PU were electroactive in PBS (100 mmol·L^−1^). So, other media were applied to try verifying the electrochemical condition where histamine would be inactive whereas the PU would be active using the identical potential range. The histamine solution was dissolved in PBS (100 mmol·L^−1^) and lithium perchlorate (0.1 M) and electrochemically analyzed. The study was applied to examine the histamine analysis using the electrode before the NIP and MIP applications were applied. The outcomes acquired are presented in Figure 3(a). The application of lithium perchlorate affected the electroactivity of histamine, which did not show at 0 to +0.6 V. On the other hand, the electroactivity of PU persisted under the parameters applied. Thereby, the application of lithium perchlorate as a media confirms the formation of PU film without influencing the histamine structure on the electrode surface.

Electropolymerization was employed to produce the MIP film. This was obtained by bulk polymerization, where the particular electrical parameters were used in a solution containing histamine as a template and PU as a polymer. This was also generating MIP material for tiny-size target analytes. The combination of electropolymerization and bulk polymerization was a worthwhile technique considering the little reagent use. In addition, this technique confirmed that imprinted areas were attached to the outer layer of the chemical sensor compared to the surface imprinting [33, 41].

In this study, a formation of PU and histamine occurred in the prepolymer arrangement owing to entangling hydrogen bond interactions. Afterward, the polymeric system was shaped by a radical reaction, which commenced by providing the adequate potential to produce oxidized radicals of PU. A chronoamperometric method was applied at a potential of +0.75 V for 100 s and was validated. A mingled solution of PU and histamine in lithium perchlorate was acquired using the electropolymerization method, generating a film over the histamine, whereas the NIP electrode acted as a control/blank and was obtained by using the PU with lithium perchlorate, so the film was acquired without the histamine. The data of CV and EIS are presented in Figure 3. In general, the presence of polymeric reactions in CV was emphasized by the anodic current reduction (Figure 3(a)), and compared to the SPE surface, the *R*_ct_ rose in the Nyquist plot as shown in Figure 3(b).

Furthermore, according to the histamine appearance in the MIP, there was a notable dissimilarity between the NIP and MIP electrodes because it was the solo experimental difference in those materials. The measurement of CV in the SPE/MIP electrode after the chronoamperometry method presented that the anodic current was more decreased and had an elevated peak separation than the SPE/NIP electrode, as shown in Figure 3(b). Thus, the appearance of histamine in the polymeric network can be confirmed by the high value of *R*_ct_ in the MIP film. The alteration of electrical properties on the surface caused by the histamine presence occurs when the polymer film grows. It can be deduced that histamine is not a conductive compound, or it can be stated that the electrical features of PU formed, particularly owing to the conductivity of PU related to its polymerization [42]. Furthermore, referring to the first stage of MIP polymerization, when the conductive polymer layer is produced, it causes the production of fewer radicals per unit of time at the outer surface. The reproducibility of electrodes was studied. Furthermore, the outcomes of CV and EIS analysis showed satisfactory reproducibility of the electrochemical approaches.

The completion of MIP installation can be done after the histamine is disposed from the polymer network. The purpose of histamine removal was to increase the binding sites for the specific analyte that would be shaped inside the polymer [43, 44]. Moreover, water can be used to dissolve histamine easily; thus, water can be categorized as a satisfactory solvent to dispose of histamine molecules from the MIP surface, so the application of other solvents can be avoided because those solvents can cause damage to the PU layer.

The incubation of MIP and NIP films in water was a condition to study the effectiveness of histamine disposal from the surface. The value of *R*_ct_ in NIP and MIP films, according to Nyquist, was escalated while the anodic peaks of the CV were reduced. It is because of the release of conductive oligomeric structures of PU from the polymeric network. Furthermore, histamine is positive when the parameter analysis is applied, thereby developing ionic interactions with the negatively charged iron redox probe and modifying the charge-transfer properties at the SPE surface. In general, the outcomes reported the effectiveness of histamine disposal on the surface of MIP and NIP films and the satisfactory stability of the PU film when unwrapped from the water.

**Figure 4:**
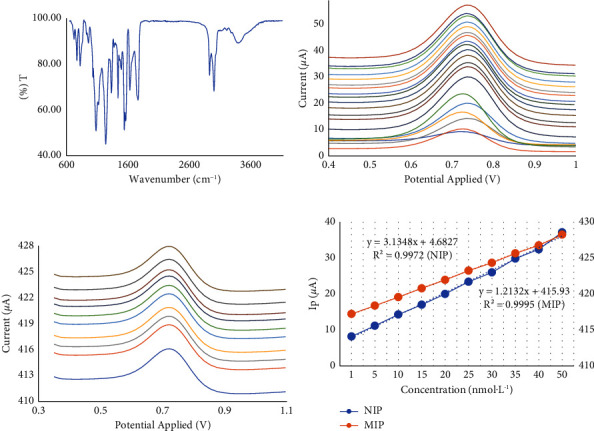
(a) The FTIR spectrum of the MIP film. (b) The voltammograms of DPV after analyzing histamine in various concentrations (1–1000 nmol) in PBS (100 mmol·L^−1^) at pH 7 on NIP/SPE/PU/LiClO_4_. (c) The voltammograms of DPV after analyzing histamine (1–50 nmol) in various concentrations in PBS (100 mmol·L^−1^) at pH 7 on MIP/SPE/PU/LiClO_4_. (d) Calibration curves of NIP/SPE/PU/LiClO_4_ and MIP/SPE/PU/LiClO_4_ between the current (*µ*A) and concentration (nmol·L^−1^) in various histamine concentrations (1–1000 nmol·L^−1^).

The application of FTIR analysis was employed in order to verify the structure of MIP film (Figure 4(a)). According to the MIP spectrum, it can be seen that there is no histamine structure, and it can be concluded that the histamine removal steps are complete and successful. Furthermore, the DPV technique was employed because it has better selectivity and sensitivity compared to the CV technique. Furthermore, this method was used to determine the oxidation of histamine during analysis using NIP/SPE and MIP/SPE. After trying various validation methods to achieve the best scan rate, pulse time, and pulse amplitude, the DPV method used 10 mV·s^−1^ as a scan rate, 50 mV as the pulse amplitude, and 10 ms as the pulse time. Various concentrations of histamine were used and analyzed using the DPV method with the validated technique.

The procedure of histamine analysis using the SPE/MIP and SPE/NIP electrodes was studied by putting the histamine standard at 100 nmol·L^−1^ on the SPE as a working electrode for 15 min, this was the incubation step, and ensuring the histamine attached to the MIP and NIP films. The determination of histamine was employed using the DPV approach on the three-electrode systems. It was replicated for several procedures, such as first escalating the histamine levels to study the electrodes and using various concentrations, starting from 1–1000 nmol·L^−1,^ and for NIP electrodes as presented in Figure 4(b). The data from DPV show the SPE/MIP electrodes that determine the blank solution and histamine levels with a series of concentrations ranging from 1–50 nmol·L^−1^, organized in lithium perchlorate and establishing a calibration curve where *x* was the histamine concentration and *y* was the values of potential applied as presented in Figure 4(c). The calibration curves between the NIP/SPE and MIP/SPE were established and are shown in Figure 4(d), showing the histamine in different levels in PBS (100 mmol·L^−1^) at pH 7. The plots of NIP/SPE and MIP/SPE result in the detection limit that has been calculated using the specific equation containing the standard deviation of the intercept and the slope of the calibration curve. The detection limit of NIP/SPE was found at 709 nmol·L^−1^, whereas the MIP/SPE was obtained at 1.765 nmol·L^−1^, respectively.

**Figure 5:**
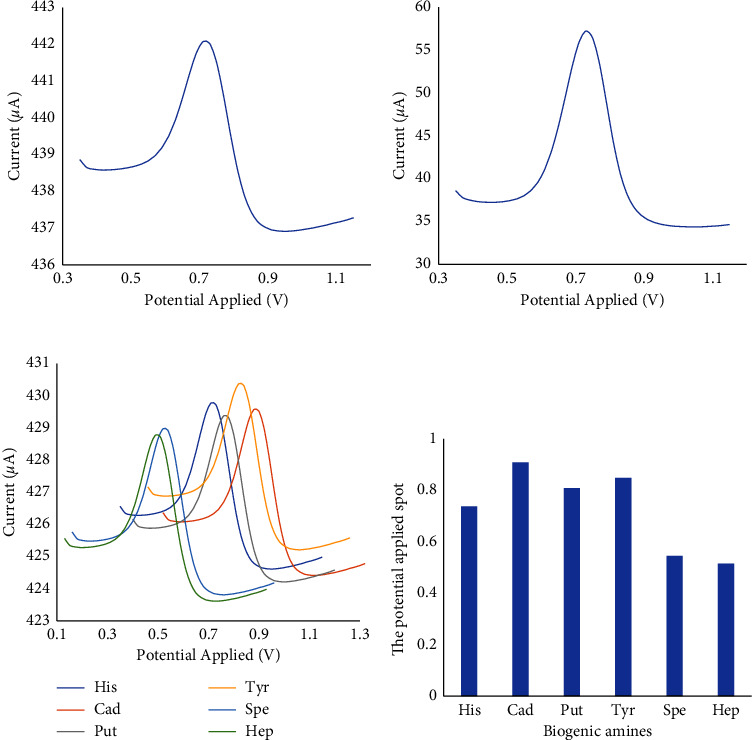
The DPV voltammograms of histamine in fish mackerel using PBS (100 mmol·L^−1^) at pH 7 on (a) MIP/SPE/PU/LiClO_4_ and (b) NIP/SPE/PU/LiClO_4_. (c) The DPV voltammograms of biogenic amines using PBS (100 mmol·L^−1^) at pH 7 on MIP/SPE/PU/LiClO_4_. (d) The selectivity plot of biogenic amines against potential applied (V).

Based on Figures 4(b) and 4(c), it can be justified that the application of MIP/SPE/PU/LiClO_4_ can increase the conductivity of the electrode, which is very suitable for this study. The further experiment in this study used MIP/SPE/PU/LiClO_4_ to analyze histamine in samples. Furthermore, the validated method was applied for histamine analysis in fish mackerel. The sample was analyzed in nine replicates and the voltammograms are presented in Figure 5.

The real level of histamine in mackerel was obtained by the known multiple standard addition technique, as the samples were spiked with a particular concentration of histamine. These analyses were made for mackerel samples exposed for several hours at ambient temperature. Furthermore, the real level in the fish mackerel was measured by interacting with the standard addition method for a logarithm response in the *x*-axis [36]. The validation of the histamine standard was performed to obtain the histamine levels in fish quantitatively. The MIP/SPE/PU/LiClO_4_ was applied to analyze histamine in fish, and the level was found at 106.32 nmol·L^−1^, whereas the NIP/SPE/PU/LiClO_4_ application determined the histamine in fish at 81.49 nmol·L^−1^. However, Figure 5(c) studied the selectivity of the electrochemical approach of MIP/SPE/PU/LiClO_4_. There were several biogenic amines were applied in this study besides histamines, such as cadaverine, tyramine, spermidine, heptylamine, and putrescine. The potential applied (V) of biogenic amines was compared, and all of them showed different spots according to Figure 5(d), thus showing satisfactory selectivity. Generally, the outcomes demonstrated that MIP/SPE/PU/LiClO_4_ was selective and able to analyze the histamine without the disturbance of other biogenic amines, although the histamine level in the mackerel was below the linear response range of the electrochemical sensor. The electrochemical sensor method may be further applied to follow up on the formation of biogenic amines and determine fish degradation.

## 4. Conclusions

This study reported the production of histamine electrochemical sensing using MIP, which is fast, simple, easily operated, and inexpensive. The difficulty of this study was obtaining the target molecule's electroactivity, which was handled by applying the experimental conditions that decreased the histamine electroactivity while maintaining the PU electroactivity. This manuscript has studied the electrochemical features of PU and histamine, whether in single or mixed solutions. It revealed that the PU electroactivity persisted under the other conditions. Furthermore, the application of a lithium perchlorate medium would ensure the possibility of forming PU film without disturbing the histamine structure. Overall, in a few seconds, the receptor element was obtained in situ; furthermore, the histamine detection on-site in an aquatic environment can be done very well, but the incubation must be applied for 15 min. The SPE/MIP instrument presented satisfactory detection in terms of analytical performance, showing high selectivity and sensitivity compared to other biogenic amines such as cadaverine and putrescine. The instrument can be applied to determine the accumulation of biogenic amines that are specifically used in the food industry.

## Data Availability

The data used to support the findings of this study are available from the corresponding author upon request.
